# Detecting and characterizing mixed infections with genetic variants of *Anaplasma phagocytophilum* in roe deer (*Capreolus capreolus*) by developing an *ankA* cluster-specific nested PCR

**DOI:** 10.1186/s13071-017-2316-0

**Published:** 2017-08-07

**Authors:** Maggy Jouglin, Sophie Chagneau, Frédéric Faille, Hélène Verheyden, Suzanne Bastian, Laurence Malandrin

**Affiliations:** 1 INRA, UMR1300 Biology, Epidemiology and Risk Analysis in Animal Health, CS 40706, F-44307 Nantes, France; 2 Bretagne-Loire University, Oniris, UMR BioEpAR, F-44307 Nantes, France; 3 CEFS, Toulouse University, INRA, Castanet Tolosan, France

**Keywords:** *Anaplasma phagocytophilum*, *Capreolus capreolus*, *ankA* variant, Co-infection, Nested polymerase chain reaction, France, Reservoir, Tick-borne diseases

## Abstract

**Background:**

*Anaplasma phagocytophilum* is a tick-transmitted Gram-negative obligate intracellular bacterium able to infect a wide variety of wild and domestic animals worldwide. Based on the genetic diversity observed with different molecular markers, several host-specific lineages have been identified. Roe deer is one of the most important reservoirs of this bacterium and hosts different genetic groups sometimes found on domestic animals. We therefore developed an *ankA* cluster-specific nested PCR (nPCR) to evaluate the prevalence of the three different *ankA* genetic groups described in roe deer (clusters II, III and IV) at three locations in France and the level of co-infections.

**Results:**

The specificity of the three nPCRs was assessed by partially sequencing 35 amplicons of *ankA* genes obtained from the different nested PCRs. All three genetic lineages were detected in roe deer from all three geographical locations. Of the infected deer population, 60.7% were co-infected by two or three different genetic variants. Co-infections varied from 42.9 to 70.6% of the infected population depending on the local infection prevalences (from 33.3 to 73.9%). All types of mixed infections occurred, suggesting the absence of a strict variant exclusion by another variant.

**Conclusions:**

Mixed infections by two or three genetic variants of *A. phagocytopilum* are a common feature in roe deer. Genetic variants (cluster IV) also found in domestic ruminants (cattle and sheep) were present in all the roe deer populations analyzed, suggesting a shared epidemiological cycle.

**Electronic supplementary material:**

The online version of this article (doi:10.1186/s13071-017-2316-0) contains supplementary material, which is available to authorized users.

## Background

*Anaplasma phagocytophilum* is a Gram-negative obligate intracellular bacterium belonging to the family *Anaplasmataceae*. Molecular phylogenetic analysis showed that the three following microorganisms (*Ehrlichia equi*, causing equine granulocytic ehrlichiosis; *Ehrlichia phagocytophila*, causing tick-borne fever of ruminants; and the human granulocytic ehrlichiosis agent -HGE-) were in fact a single species [1].

As a single species, *Anaplasma phagocytophilum* displays noticeable diversity and versatility. It infects a large range of wild and domestic animal species worldwide (birds, reptiles, mammals) and is transmitted by different tick species depending on the geographical area, but mainly of the genus *Ixodes* [2]. It is therefore able to invade and multiply within the granulocytes of diverse vertebrate hosts, as well as within different cell types of its tick vector (tick gut and salivary gland cells). Apparently this bacterium has developed common molecular strategies for infection, compatible with its heavily reduced genome [3]. Even so, vertebrate host tropism, pathogenicity and clinical manifestations seem to vary between different *A. phagocytophilum* strains, and imply the existence at some stage of specific infection strategies, which still remain largely unknown.

*Anaplasma phagocytophilum* is also genetically diverse, and genetic variants from various geographical and host origins have been characterized. The markers most frequently used to assess this variability are the 16S rDNA, the *groESL* operon, the outer membrane protein genes (*msp2* and *msp4*) and *ankA* [4, 5]. At least 15 variants, differing in a 10 bp 5′-variable fragment of the 16S rDNA, have been described, but more variants can be discriminated based on nucleotide substitutions spread over more conserved regions of the gene [4, 6, 7]. Although this marker was reported to be able to discriminate between human pathogenic and non-pathogenic variants in the USA [8], the situation is not as clear in Europe where no host species segregation could be confirmed [4, 6, 7]. Four ecotypes have been defined, based on *groESL* sequences from European *A. phagocytophilum* variants collected from diverse hosts [9]. While the host ranges are well defined for ecotypes III (rodents) and IV (birds), 68% of the ecotype I samples have very diverse host origins (including humans), and ruminant variants are found in both ecotypes I and II of this marker. *msp2* belongs to a multigene superfamily with antigenic variation resulting from combinatorial gene conversion into an expression site, and is therefore not a candidate of choice for phylogenetic analysis. Moreover, despite the high level of polymorphism, *msp2* sequences have never clustered by countries, hosts or vector ticks [10–13]. On the other hand, *msp4* is a single copy gene, and most *msp4* haplotypes usually group into a clade whatever the host of origin (human, horse, dog, red and roe deer, sheep, bison, wild boar, bear, bird or wood rat), except the rodent variants that segregate into a separate clade [14–16].

The value of *ankA* as a highly polymorphic and discriminatory marker was suggested in different studies analyzing *A. phagocytophilum* from ticks or diverse hosts [17–20]. The correlation with host species was demonstrated in a comprehensive study of 198 complete *ankA* sequences from nine hosts [6], and an additional study focusing on voles and shrews [21]. Five clearly distinct clusters supported by bootstrap values of at least 98% were delineated. Human (Europe and USA), dog, cat and horse sequences were grouped exclusively in cluster I, and rodent sequences exclusively in cluster V. Even though the ruminant sequences were distributed in clusters I to IV, clusters II and III were mainly restricted to roe deer. The list of hosts infected with the cluster I variants of *A. phagocytophilum* now includes a wide range of species (human, dog, cat, horse, sheep, cattle, goat, red deer, bison, chamois, wild boar, bear, hedgehog and red fox [6, 16]. However, these studies may have a geographical bias as most of the sequences were from Europe [6, 7]. New clusters might be defined if samples from other parts of the world were analyzed. Two types of multilocus sequence analysis were used to type *A. phagocytophilum*: multilocus sequence typing (MLST) [7] and multilocus variable number tandem repeat analysis (MLVA) [22]. The MLST analysis differentiated 90 sequence types (284 strains, 16 host species), forming eight clonal complexes with 41 unlinked sequence types. As with the results obtained solely with the *ankA* marker, MLST revealed a strong relationship with the host and geographical origin [7]. The MLV analysis, based on five intragenic minisatellites, was highly discriminatory and detected 84 profiles among 125 DNA samples from a more restricted host range (7 host species) [22].

In these studies and using different markers, *Anaplasma phagocytophilum* sequences from rodents on the one hand, and from humans, horses and dogs on the other hand, were separated into two clusters or clonal complexes. The situation regarding ruminant sequences is far more complex, since they are dispersed among different clusters, including the clonal complex that features sequences from human samples [6, 7, 9, 21]. Depending on the marker used, *A. phagocytophilum* sequences from roe deer can be grouped into 3 *ankA* clusters (II, III and IV) [6], into 2 *groEL* ecotypes (I and II) [6, 7, 9, 21], into 11 MLST sequence types most of them unique and unlinked to any clonal complex (among 12 sequences analyzed) [6], or into seven MLVA profiles (15 sequences analyzed) [22]. Vertebrate hosts are considered to serve as critical reservoirs in the life-cycle of *A. phagocytophilum*, since transovarial transmission may occur in *Ixodes* tick species, but at a low level [9, 23]. Whether roe deer are involved in epidemiological cycles likely to involve the infection (even accidental) of humans or livestock remains to be clarified [7, 24]. In roe deer, prevalences of *A. phagocytophilum* higher than 70% have been reported in several European countries (Germany, France, Slovenia and Austria) [6, 24–28]. With such high prevalences, co-infections or superinfections are therefore likely to occur [29]. They represent a major drawback for multilocus sequence analysis as noticed on large wild animals such as roe deer [7].

Our aim in the present study was to develop a typing nested PCR (nPCR) for *A. phagocytophilum* from roe deer to: (i) analyze the occurrence of co-infections or superinfections with different genetic variants in this host species; and (ii) help clarify its role as reservoir of strains involved in different epidemiological cycles shared potentially with domestic ruminants such as cattle or sheep. We chose *ankA* as a marker as it separates roe deer restricted variants (clusters II and III) from multi-host (ruminants) variants (cluster IV). *ankA* is also known as being a highly polymorphic gene (more than 50% of polymorphic sites) [6, 30], that exhibits a strong association with the host of origin (comparable to MLST) [6, 7]. We developed three *ankA* cluster-specific nPCRs, two for the *ankA* clusters usually described from roe deer (II and III), and one for the *ankA* cluster shared with domestic ruminants (cluster IV). We also setup a species-specific nPCR to detect a possible circulation of other cluster variants.

## Methods

### Sample collection

This study was conducted in either captive or free-ranging roe deer from three sampling sites in France. In Brittany, roe deer were hunted in the Villecartier forest, Ille et Vilaine (48°28′26″N, 1°33′29″W). Spleens from 21 and 25 roe deer were collected during the winters of 2014 and 2015, respectively, and frozen at -20 °C until used.

In Haute-Garonne in the south of France, blood samples were obtained from roe deer at two locations: the Aurignac site (43°13′10″N, 0°52′49″E) and the INRA experimental station at Gardouch (43°23′31″N, 1°41′04″E). The dynamics and ecology of a wild roe deer population is being studied at the Aurignac site since 2000. In January and February, roe deer are driven by beating into nets placed at six capture sites. At the INRA experimental station, some roe deer are raised in 0.5 ha enclosures and fed with pelleted food, whereas others are free-ranging in a large enclosure (9 ha of woodlot and 5 ha of meadow) without artificial feeding. Roe deer on the experimental station are caught once a year to monitor their health status. Once captured, each roe deer is tranquilized by intramuscular acepromazine injection (0.3 ml), maintained quietly in a wooden box for 30–300 min before being manipulated at the end of the capture session. The roe deer are manipulated by experienced, authorized people, in accordance with the European directive (2010/63/UE) for care and use of animals (agreement N° A31113001 and A31210001). In all cases, blood samples were collected from the jugular vein and anticoagulated blood samples were kept at 4 °C before treatment.

### DNA extraction

The 46 frozen spleens were cut at three different points (center and extremities) and 50 mg from each piece were combined, shredded, and incubated overnight at 56 °C with 300 μl lysis buffer T1 (Macherey-Nagel, Düren, Germany) and 50 μl proteinase K solution (20 mg/ml) before DNA extraction. Genomic DNA was extracted from the spleen with the NucleoSpin Tissue kit (Macherey-Nagel, Germany), according to the manufacturer’s instructions.

The 61 whole blood samples were centrifuged, the plasma was discarded and the remaining cells (buffy coat and red blood cells) were washed with PBS (phosphate-buffered saline) and stored in PBS at −20 °C until used. Genomic DNA was then extracted using a genomic DNA extraction kit (Wizard Genomic DNA Purification Kit, Promega, Madison, USA), according to the manufacturer’s instructions.

### Selection of primers specific to *ankA* clusters II, III and IV

To select primers conserved within each *ankA* cluster but discriminating the *A. phagocytophilum* variants according to their clusters, the 5′ region of the *ankA* gene was selected, from nt 1 to 1650, as a highly variable region of the gene but outside the region where recombination has been previously reported [7, 21]. *ankA* sequences covering the selected region and belonging to the 5 clusters were selected from the studies [6, 7, 21]. They were then retrieved from GenBank, aligned using clustalomega and a consensus sequence (highlighting conserved and variable positions) was established for each cluster. The cluster I consensus sequence was established by aligning 95 *ankA* complete sequences from 43 dogs, 12 humans, 12 sheep, 10 horses, 9 European bisons, 5 red deer, 3 cows and 1 cat [6], and 30 partial sequences (523 bp) from hedgehogs [7]. Consensus sequences for clusters II, III and IV were based on 31 sequences from roe deer, 12 sequences from roe deer and red deer and 59 sequences from diverse ruminants (sheep, red deer, roe deer, cow and European bison) respectively [6], while 27 *ankA* sequences from rodents were aligned for cluster V [21]. By comparing the 5 consensus sequences, *A. phagocytophilum ankA* primers were designed to amplify a 1609 bp fragment of the clusters I to IV variants (nPCR external primers). Internal primers were then designed to either specifically amplify clusters II, III and IV variants, or all possible variants from clusters I to IV as a control (Ana-Int-F1 and Ana-Int-R3) (Table 1).

**Table 1:** List and characteristics of primers designed in this study

Primer type	Primer name	Sequence 5′–3’	Cluster specificity^a^	Melting temperature (°C)	Amplicon size (bp)
External primers	Ana-Ext-F2	TCTGMAAGCCATTATCAYAG	I, II, III, IV	58	1609
	Ana-Ext-R1	TGCTTCACGAARCGCATA			
Internal primers	Ana-Int-F1	CATCKAGCAKGTRTTGAAGG	I, II, III, IV	58	731–737
	Ana-Int-R3	TGTARAGGRGCARMACC			
Sequencing primers	Ana-Int-F2	GCGAGTGTGCAGASKCACTA	I, II, III, IV	58	328
	Ana-Int-R3	TGTARAGGRGCARMACC			
Internal cluster-specific primers	Ana-F-Cl2	GTGACAATTTTGRGACATTAC	II	60	1512
	Ana-R-Cl2	TCCTAGTCCGCGAGCTAAGT			
	Ana-F-Cl3	GTGAGAATTTTGAGTCATTG	III	60	1512
	Ana-R-Cl3	TCCGAACCCGRGCGCTCAT			
	Ana-F-Cl4b	GAGGTATCTAGGAAGTGCC	IV	61	1286
	Ana-R-Cl4	TCAAGTTCCGTGCGGTTAGG			

^a^Cluster numbers as in [6]

### PCR analyses and sequencing

To perform the first PCR, 5 μl of genomic DNA was used as a template in a final volume of 30 μl containing 0.33 mM dNTPs (Eurobio, France), 1X PCR buffer, 2.5 mM MgCl_2_, 1 μM of each primer, 0.2 μl (1 U) of GoTaq Flexi (Promega) and deionized water. PCR steps included 95 °C for 5 min - 40 cycles of 30 s at 95 °C, 30 s at the selected annealing temperature (Table 1), 30 s at 72 °C - and a final elongation step of 5 min at 72 °C. Four nested PCRs were run (one for each cluster II, III and IV, and one with cluster I-IV primers), using 10 μl of the first amplicon (diluted 1:100) as template. Presence and sizes of the amplicons were controlled under UV on an ethidium bromide-stained 1.5% agarose gel.

To control the specificity of the cluster-specific nested PCR, amplicons were purified using ExoSAP-IT (Ozyme, Saint Quentin en Yvelines, France) and sent for Sanger conventional sequencing in both directions (GATC, Germany). Conserved internal primers (Ana-Int-F2/Ana-Int-R3), framing a highly discriminatory region between clusters (position from nt 854 to 1182), were specifically designed to allow cluster assignment.

### Accession number of nucleotide sequences

A selection of 10 *ankA* partial sequences, representing each different variant and the different geographical regions, was deposited in GenBank (KY618918–KY618927). For example, all sequences for cluster IV were identical so only one was deposited (KY618927). Sequences of 4 variants of cluster II can be found under accession numbers KY618918- KY618921, as well as sequences of 5 variants of cluster III (KY618922- KY618926). For details about the variants, see Additional file 1: Table S1.

## Results

### Prevalence of *A. phagocytophilum* in roe deer at three locations in France

A roe deer sample was considered positive as soon as one positive amplicon was obtained in the four possible nPCR. *Anaplasma phagocytophilum* was detected in 56/107 roe deer (52.3%) from all three sites, with variable local prevalences. Prevalences were higher in north-west France, at Villecartier, when spleens from free hunted roe deer were analyzed (73.9%; 34/46), than in blood samples taken in south west France (33.3%; 7/21 in Gardouch and 37.5%; 15/40 in Aurignac). At the experimental station at Gardouch, prevalence was higher in roe deer from the large enclosure (71.4%; 5/7) than in animals kept in the small enclosure with food supplementation (14.3%; 2/14). In the Villecartier forest in Brittany, where spleens from hunted roe deer were analyzed for two successive years, the prevalence remained stable, with 76.2% (16/21) in 2014 and 72% (18/25) in 2015.

### Sequencing control of amplification specificity

To confirm the specificity of the primer sets, especially between-cluster discrimination, a subset of 35 amplicons was sequenced (314 bp). This subset was chosen to represent sampling diversity, i.e. either from blood (*n* = 19) or spleen (*n* = 16), from different roe deer (*n* = 30) from the three different locations and collected in two different years. Sequences were compared to the GenBank database by applying the Blastn algorithm. Sequence identities within *ankA* cluster were defined previously as being always higher than 95%, while identities between clusters droped below 85% [6, 7]. Cluster assignation of the sequences blasting with identities higher than 95% was controlled in each corresponding papers.

To control the nPCR specificity, amplicons were sequenced either from roe deer with apparent mono-infection (only one cluster-specific nPCR was positive) or apparent mixed infections (two or three cluster-specific nPCRs were positive). Nine amplicons obtained using cluster II-specific primers were sequenced, 14 amplicons from cluster III-specific nPCR and 12 amplicons from cluster IV-specific nPCR. The Blastn confirmed the specificity of the amplification.

For 5 roe deer, the presence of multiple variants in the same animal was confirmed by sequencing the different amplicons. All these controls validated the cluster specificity of the newly designed primers and their ability to detect mixed infections with multiple *ankA* variants.

### Proportion of *A. phagocytophilum* variants in roe deer

**Fig. 1:**
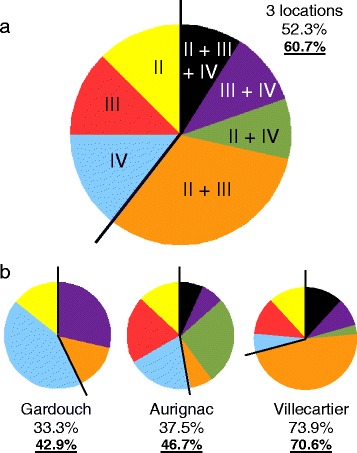
Prevalences of mono- and mixed-infections by *A. phagocytophilum ankA* variants in roe deer. **a** Global analysis of the three geographical locations in France. **b** Analysis of each location separately. In each case, the first value corresponds to the infection prevalences, the second value (bold and underlined) to the percentage of mixed infections in the population of infected roe deer

Ninety-five different amplicons were found in the 56 infected roe deer but only *ankA* variants belonging to the three previously described roe deer clusters (II, III and IV) were detected. No circulation of variants from cluster I could be demonstrated. The overall proportions of variants among the 95 amplicons were of 36.8% for cluster II, 37.9% of cluster III and 25.3% of cluster IV, which were not significantly different (Chi-square test, *χ* ^2^ = 2.8, *df* = 2, *P* = 0.25). Variants from each cluster were detected in all three geographical locations, with differences between sites. Noteworthy was the significantly lower proportion of cluster IV variants in the Brittany samples from Villecartier forest (16.1%; Chi-square test, *χ* ^2^ = 8.3548, *df* = 2, *P* = 0.01534) (Table 2 and Fig. 1).

**Table 2:** Prevalence of *ankA* genetic variants in three geographical locations in France. As mixed infections occured frequently, 95 *ankA* genetic variants were detected in the population of 56 infected roe deer

Cluster^a^	Three locations	Gardouch	Aurignac	Villecartier
Cluster II	35/95 (36.8%)	2/10 (20%)	8/23 (34.8%)	25/62 (40.3%)
Cluster III	36/95 (37.9%)	3/10 (30%)	6/23 (26.1%)	27/62 (43.6%)
Cluster IV	24/95 (25.3%)	5/10 (50%)	9/23 (39.1%)	10/62 (16.1%)

^a^Cluster numbers as in [6]

### Prevalence of *A. phagocytophilum* mixed infections in roe deer

Mixed infections were detected in 60.7% of the 56 infected roe deer. The percentage of mixed infections varied depending on the geographical location: 42.9% in Gardouch, 46.7% in Aurignac and 70.6% in Villecartier (Fig. 1), increasing with the prevalences of infection (33.3, 37.5 and 73.9%, respectively).

Variants from all three clusters were detected as single as well as mixed infections, and all combinations of mixed infections occurred, including triple infections (Fig. 1 and Table 3). This result indicates an apparent absence of variant exclusion when roe deer are infected. The prevalence of the different cluster combinations detected in mixed infections varied with the location. In Villecartier, the high prevalence of the mix of clusters II + III (47.1% of the locally infected roe deer) correlates with the considerable local circulation of these variants detected as a single infection. This was not the case in Aurignac where the low prevalence of mixed infections with variants from clusters III and IV (6.7%) contrasted with the high prevalence of these clusters as single infections (20% each). The same discrepancy was noted at Gardouch with clusters II and IV. This poor representativity could be explained by the few positive samples in these last two locations (15 and 7, respectively).

**Table 3:** Prevalences of cluster mono- or mixed-infections and prevalences of cluster combinations in the 56 roe deer infected population

Cluster^a^	Mono-infections	Double infections		Triple infections
		+ III	+ IV	+ III + IV
Cluster II	**7/56 (12.5%)**	**18/56 (32.2%)**	**5/56 (8.9%)**	**5/56 (8.9%)**
	G: 1/7 (14.3%	G: 1/7 (14.3%)	G: 0/7 (0%)	G: 0/7 (0%)
	A: 2/15 (13.3%)	A: 1/15 (6.7%)	A: 4/15 (26.7%)	A: 1/15 (6.7%)
	V: 4/34 (11.8%)	V: 16/34 (47.1%)	V: 1/34 (2.9%)	V: 4/34 (11.8%)
Cluster III	**7/56 (12.5%)**		**6/56 (10.7%)**	
	G: 0/7 (0%)		G: 2/7 (28.6%)	
	A: 3/15 (20%)		A: 1/15 (6.7%)	
	V: 4/34 (11.8%)		V: 3/34 (8.8%)	
Cluster IV	**8/56 (14.3%)**			
	G: 3/7 (42.8%)			
	A: 3/15 (20%)			
	V: 2/34 (5.%)			
Total	Mono-infections	Double infections		Mixed infections (double and triple)
	**22/56 (39.3%)**	**29/56 (51.8%)**		**34/56 (60.7%)**
	G: 4/7 (57.1%)	G: 3/7 (42.9%)		G: 3/7 (42.9%)
	A: 8/15 (53.3%)	A: 6/15 (40%)		A: 7/15 (46.7%)
	V: 10/34 (29.4%)	V: 20/34 (58.8%)		V: 24/34 (70.6%)

*Abbreviations: G* Gardouch, *A* Aurignac, *V* Villecartier

^a^Cluster numbers as in [6]. In each case, the first line in bold characters corresponds to the three locations together, and the three following, to each location separately

### Genetic diversity of the sequenced variants

Sequence alignments (314 bp) of variants from the same cluster revealed intracluster genetic diversity for clusters II (comparison of 9 sequences) and III (comparison of 14 sequences) (Additional file 1: Table S1), but not for cluster IV (comparison of 12 sequences from the three different study sites). Despite the low level of intracluster diversity (5 substitutions/314 bp, 1.6%), four variants were distinguished in cluster II, three of them unique, and one shared by six samples from the three different study sites (Additional file 1: Table S1). The most frequent variant of cluster II detected in our study (6 different samples) has already been detected in infected roe deer from different European countries (Slovenia, Spain and Germany) [6], while two variants remain unique to our study.

The 14 sequences from cluster III were separated into five variants (6 substitutions/314 bp, 1.9%), only one of them being unique (Additional file 1: Table S1). Three of them had already been described in *A. phagocytophilum* infected roe deer in Germany, but the other two had not yet been described [6].

Whatever the cluster, the variants detected in our study were apparently not linked to the geographical location of the infected roe deer.

## Discussion

The prevalences of *A. phagocytophilum* in roe deer obtained in the European Community ranged from 9.6 to 98.9% [2]. We found in France an overall prevalence of 52.3% when the three geographical sites are considered. Prevalences of 78 and 95.2% were reported in south-west France and central France, respectively, after qPCR of the *msp2* or *p44* gene [24, 30]. Our results confirm the role of roe deer as an important natural reservoir of *A. phagocytophilum*.

In our study, a great variability of prevalence was observed according to the sampling site. The lower prevalence detected in the small enclosure in Gardouch (14.3%) could be explained by the fact that the sampled animals were captive-bred roe deer kept in a restricted environment unfavorable to tick maintenance. A lower prevalence of infected farmed breeding red deer, as compared to wild cervids, has already been documented [31]. A probably lower abundance of *I. ricinus* ticks in enclosed habitats as well as a better health status of these animals could result in a lower infection rate of tick-transmitted pathogens and more efficient clearance of the pathogens, respectively. The highest infection prevalence was recorded in Villecartier, when spleens were analyzed. Whether this higher prevalence was related to the sample type (spleen instead of blood) or to real differences in prevalence could not be determined, since the two types of sample were not obtained from the same animals. In publications, the conclusions drawn after comparative analysis of the infection rates in spleen or blood from the same animals can differ: infection rates may be similar [25], or detection may be better in blood [32, 33] or in spleen samples [34]. As many factors can vary from one study to another (type of anticoagulant, inhibitors from blood such as hemoglobin, DNA extraction protocols, primers used, etc.) and interfere with PCR detection [35], it is difficult to conclude which sample type is the most suitable.

The specificity of the designed *ankA* cluster-specific nPCR was confirmed by sequencing 35 amplicons obtained with the four sets of primers (one non-cluster-specific set and three cluster-specific sets) either from an apparent single infection (only one amplicon with one of the three specific amplifications), or from apparent mixed infections (amplicons obtained separately from two or all three specific nPCRs) to ensure the absence of cross amplifications when template DNAs are mixed. Thus, the designed nPCR protocol allows the specific amplification of *ankA* variants from clusters II, III and IV. In our study, variants from these three clusters were detected at all three geographical locations in France. Variants from clusters II and III were slightly more abundant than variants from cluster IV, but their relative proportions depended on the geographical location of the roe deer. For example, the prevalence of cluster IV variants fluctuated between 16.1% (Villecartier) and 50% (Gardouch). In previous studies, variants from clusters II and III were clearly more abundant than variants from cluster IV in roe deer (data from [6] and [7] respectively), cluster II variants being identified in all European animals sampled (in Germany, Spain, Norway, Slovenia and France) [6, 30]. According to these studies, cluster III variants were detected in roe deer in Germany and France, but the cluster IV variants present in Germany, Norway and Slovenia could not be detected in roe deer in France, even in Haute Garonne [30], while it was the main cluster in our study (42.4% of the variants). Its occurrence may have been missed in the latter study due to the use of a pre-amplification of DNA (multiple displacement amplification) followed by a cluster non-specific nPCR. The low bacterial load and high occurrence of mixed infections in asymptomatic roe deer (which reduced the load of each variant) may have resulted in amplification of the numerically more abundant variant. The same protocol used in the same study on cattle blood samples indicated a high prevalence of cluster IV variants (49.5%), thus excluding a primer selectivity effect. However, the authors analyzed blood from clinically sick cattle (higher bacterial load) with probably one dominant variant in the acute phase. The unidirectional suppression of genotypes has indeed been observed in experimental infections of lambs following their simultaneous inoculation with mixtures of genetically different isolates [36]. Only 4.4% of ambiguous sequences, most probably corresponding to mixed infections, were indeed detected in cattle, compared to 19% in roe deer [30]. The presence of cluster IV variants in 42.9% of the infected roe deer (24/56) emphasizes the potential epidemiological role of roe deer in spreading this genotype which is found in about half of the sick cattle in France. Variants of cluster I, with a suspected role in human anaplasmosis, were not detected in roe deer when cluster I-IV amplification was performed, indicating that roe deer is probably not a reservoir for human anaplasmosis, as already suspected [6, 7].

In our study, the extent of mixed infections in roe deer could be determined by using primers specific to the *ankA* cluster. As already mentioned [7], mixed infections of *A. phagocytophilum* are more the rule than the exception, and varied from 42.9 to 70.6% in our study. This local variation was most probably linked to the local bacterial prevalences (from 33.3 to 73.9%). It seems reasonable to suppose that the higher the prevalence, the higher the rate of transmission by ticks, and thus the increased risk of mixed infections. Whether mixed infections resulted from the simultaneous transmission of multiple variants by one tick, or from super-infection (i.e. the successive transmission of variants by different ticks), or from both, remains to be elucidated. All possibilities probably occur in the wild, with animals in forest habitats particularly exposed to tick bites. This high prevalence of mixed infections, with all four possible cluster combinations detected (II + III, II + IV, III + IV and II + III + IV), implies that variant suppression, if it occurs in roe deer, is not a rapid process. Unidirectional suppression of one variant had been demonstrated in lamb experimental co-infections. However the variant detection method (non-specific and therefore leading to preferential amplification of the more abundant variant), as well as the fact that the “suppressed” variant could be detected later during the infection in 4/6 of the infected animals [36], indicated that genetically different variants may co-proliferate within the same animal and persist. In single infections, each of three cluster variants represented about one third of the mono-infected roe deer, which also suggests that all these variants are identically “adapted” to roe deer. The detection of variant IV in the two enclosed roe deer populations at Gardouch, with no possible contacts with cattle or sheep also carrying cluster IV variants, strongly indicates the ability of roe deer alone to maintain this genetic variant population. However, evaluation of the variant prevalences in co-infections revealed the under-representation of variant IV (47% of co-infections involved cluster IV variants compared to about 84% for clusters II or III). Due to the limited number of mono-infected (*n* = 22) and co-infected roe deer (*n* = 34 with 4 possible combinations), and the unequal prevalences of cluster variants in the different geographical locations studied, it is difficult to draw any conclusions about a potential exclusion of variants in the case of mixed infections.

Despite the short region sequenced to control cluster specificity of the amplification, sequence variations were observed and several variants could be distinguished within clusters II and III (4 and 5 respectively), five of them being identical in this region to other European *ankA* sequences of *A. phagocytophilum* from roe deer. Even though 12 sequences were compared, no sequence variations could be detected in the cluster IV *ankA* sequences. The unique 314 bp sequence was identical to four sequences of the *A. phagocytophilum ankA* gene obtained only from roe deer in Germany (GU236878, GU236885, GU236902 and GU236906) and differed by at least 6 nt from the other *ankA* cluster IVc sequences obtained from cattle (7 nt), bison (6–11 nt), sheep (8 nt) or red deer (9–21 nt) [6]. Whether this strong conservation relates to a strong roe deer host specificity of the *A. phagocytophilum* genetic variants, even within a cluster, remains to be clarified.

## Conclusions

In this study, we developed three nested PCRs that allowed separate detection of the three *A. phagocytophilum ankA* genetic variants present in roe deer, i.e. variants of clusters II, III and IV. When applied to 107 roe deer from three different geographical locations in France, all three types of variant, all three possible double-infection combinations, as well as infections with the three different genetic variants, could be detected in each location. The high level of co-infections in roe deer (60.7%) may have masked the presence of cluster IV variants in this animal, and our result emphasizes the need for more epidemiological research to clarify the role of roe deer as an *A. phagocytophilum* infective reservoir for cattle. The tools developed in this study could also be valuable in detecting ticks infected with multiple *A. phagocytophilum* variants.

## Additional file

Additional file 1: Table S1. Polymorphism of *A. phagocytophilum ankA* sequences within clusters II and III. Only sequences originating from roe deer samples, and in the gene region sequenced in the present study, are compared. The different sequence types are represented. The locations of the amplified region and the substitutions are presented on one of the complete sequences published by [6]. (DOC 30 kb)

## Abbreviations

*ankA*: ankyrin A gene
