# Supplementary material for: Detecting and characterizing mixed infections with genetic variants of Anaplasma phagocytophilum in roe deer (Capreolus capreolus) by developing an ankA cluster-specific nested PCR
Source: Parasit Vectors. 2017 Aug 7;10:377. doi: 10.1186/s13071-017-2316-0 (PMC5547487; doi:10.1186/s13071-017-2316-0)
Supplement: Additional file 1: Table S1. — Polymorphism of A. phagocytophilum ankA sequences within clusters II and III. Only sequences originating from roe deer samples, and in the gene region sequenced in the present study, are compared. The different sequence types are represented. The locations of the amplified region and the substitutions are presented on one of the complete sequences published by [6]. (DOC 30 kb) [file 13071_2017_2316_MOESM1_ESM.doc]

**Additional file 1: Table S1**. Polymorphism of *A. phagocytophilum ankA* sequences within clusters II and III. Only sequences originating from roe deer samples, and in the gene region sequenced in the present study, are compared. The different sequence types are represented. The locations of the amplified region and the substitutions are presented on one of the complete sequences published by [6].

*ankA* CLUSTER II

Sequence Nucleotide position Country^b^ Deposited

reference from start site^a^ in Genbank

**895** 848 **978** **981** 1099 **1103** 1105 **1110** 1146 (accession number)

CVD15-035 G C A A A G A G T France (Aurignac) KY618921

CVD15-077 A C C A A A A G T France (Villecartier) KY618920

GU236894.1 A C A A A A A A T Germany

GU236872.1 A C A A A A A A T Germany

CVD14-063 A C A A A A A A T France (Villecartier) KY618919

GU236910.1 A C A G A A A A T Slovenia

GU236908.1 A C A G A A A A T Slovenia

GU236905.1 A C A G A A A A T Slovenia

GU236903.1 A C A G A A A A T Slovenia

GU236897.1 A C A G A A A A T Spain

GU236889.1 A C A G A A A A T Germany

GU236888.1 A C A G A A A A T Germany

GU236886.1 A C A G A A A A T Germany

GU236881.1 A C A G A A A A T Germany

GU236880.1 A C A G A A A A T Germany

GU236877.1 A C A G A A A A T Germany

GU236869.1 A C A G A A A A T Germany

GU236868.1 A C A G A A A A T Germany

CVD15-003 A C A G A A A A T France

CVD15-013 A C A G A A A A T France

CVD15-043 A C A G A A A A T France

CVD15-050 A C A G A A A A T France (Gardouch) KY618918

CVD15-075 A C A G A A A A T France

CVD15-092 A C A G A A A A T France

GU236879.1 A C A A A A A A C Germany

**GU236909.1^c^ A C A R G G A G T Slovenia**

**GU236900.1^c^ A C A G A G A G T Spain**

GU236907.1 A C A G A G A G T Slovenia

GU236883.1 A C A G A G A G T Germany

GU236899.1 A C A A A G A G T Spain

GU236874.1 A C A A A G A G T Germany

GU236904.1 A T A G A G G A T Slovenia

GU236901.1 A T A G A G G A T Norway

GU236898.1 A T A G A G G A T Spain

GU236892.1 A T A G A G G A T Germany

GU236867.1 A T A G A G G A T Germany

GU236865.1 A T A G A G G A T Germany

^a^ Positions with substitutions in sequences issued from this study are noted in bold underlined

^b^ From Scharf et al., 2011 and this study (France)

^c^ These two sequences differ by a 9 bp deletion between nt 1074 and 1087

>GU236905.1 *Anaplasma phagocytophilum* isolate roe deer 472 ankA partial sequence (Scharf et al., 2011)

ATGTTGACAGAAGAAGAAACTAGAAAGAGCAAAGGTGCTCTGAAAGCCATTATCACAGGAGATTGTGACA

ATTTTGAGACATTACTTCAGGGAATTTCTACCGAAGGACTTAATACTCAAGTTGATAATAATGGCAGAAC

ATTACTGCACTATGCAGCTACGTCTCGTAATGAAAATTTCTATAACATTCTGGTTGAAAAGGGATGTGAT

GCTAATATTAAAGATGCTAACGGAATTGATTCACAGCAAGCACGTGATAAAGCGAGACGTGCTCGTACTC

AGTGGTATGGAGCAGATATAAATGATCCAAATGTAGGTAGGGCTTGCATGACGCAAGCTGTTGAGCAGTC

TGCAAAAGGTAGAGTGTATGCTGCTCTCGCTTTATTGGACCTTGCACGTAACGACGATGCAAACATGCAG

CTCAATGAGTACGGGCACAGTGTTTTGCATCTAGCATGTGTTGAAGGCAGTGATCCAGCTTTCACTGCAG

CCCTTCTGATGAAGGGTTGTTCTTTAGGGAGTAAGGATATAGATGGTAATACTCCATTACATACAGCTGC

GTTTACAGTAGGCAAAAATGCTTTAAGCAATCTTGAGGTTCTATGCGATCAATCTCTTATAGTAGATGTT

AATGCTCAGAACAAGAATGGAAACACTCCGCTGCATATTGCTACTGAGCGTATGGATCACGAGAAAATAG

ACTCTCTTATCTCGAGGTTTAGCGATATTAGTGTGGCAAATAATGCTGGTCAAAGCGTTTTCCACATTGT

TGCAGAGCGCTGGCCAAGGCGAGGGATTTCACAATATATTGAGAAAGTGCAAAAAGCGGTGTCGTCAAAT

ATTGAGGGCGAGCGCGAGTGTGCAGAGGCACTAATATTCCCAGATCAAAAAGGGRTAAGTGCAGTGCAGT

ATGTTCTTAGAAGAAATGTATCGGACGCTGGGAAGATYTTTGATACAGCTATTAACATTGCGGATAAMGT

RTACAGCTCAGGTTCCCCAGAAGTAAAATCTCTCTTCACATGCCCTGGTGCAGAGGATGCAAGAACGCTA

TTGCATTTGGTATCTTCTAATGATAGCCAAAATTTTGATCCTGTTGCGVAAARARTATTARAAGAAGCTT

ATCATAGGTTTGGAACAGAACCTTTYACTCATGTCGACATTTCAGGTAATGCACCTATACA

Start codon

Sequencing primers

Deletion (sequences GU236900 and GU236909)

Substitution

***ankA* CLUSTER III**

Sequence Nuleotide position Country^b^ Deposited

reference from start site^a^ in Genbank (accession number)

**897** **956** **1020** **1061** **1100** 1120 **1121**

CVD15-083 G C G C T T A France

CVD15-095 G C G C T T A France (Villecartier) KY618922

CVD14-054 G C G C G T A France

CVD15-091 G C G C G T A France

CVD15-050 G C G C G T A France (Gardouch) KY618923

CVD15-045 G C G C G T A France

CVD15-046 G C G C G T A France

CVD15-022 G C G C G T A France

GU236893.1 G C G C G T A Germany

GU236866.1 G C G C G T A Germany

CVD14-050 G C A T G T A France (Villecartier) KY618924

CVD15-078 G C A T G T A France

CVD14-055 G C A T G T A France

GU236895.1 A G A T G T G Germany

GU236891.1 A G A T G T G Germany

GU236882.1 A G A T G T G Germany

CVD15-023 A G A T G T G France

CVD15-094 A G A T G T G France (Villecartier) KY618926

GU236884.1 G C A T G T A Germany

GU236875.1 G C A T G T A Germany

GU236871.1 G C A T G T A Germany

GU236896.1 G C A T G T A Germany

CVD15-012 G C A T G T A France (Aurignac) KY618925

GU236887.1 A G A T G C A Germany

^a^ Positions with substitutions in sequences issued from this study are noted in bold underlined

^b^ From Scharf et al., 2011 and this study (France)

>GU236895.1 *Anaplasma phagocytophilum* isolate roe deer 30 *ankA* partial sequence (Scharf et al., 2011)

ATGTTGACAGAAGAAGATATAAAAAAGACCAAAGGTATTCTGAAAGCCATTATCACAGGAGATGGTGAGA

ATTTTGAGTCATTGCTTCAGGGAGTATCTACCGAAGTACTTAATACTCCCGTTGATAATAGTGGCAGAAC

ACTACTGCACTATGCAGCTACGTCTCGTAATGACACTTTCTATAACATTCTGGTTGGTAAGGGATGTTAC

ACTAATGTTAAGGATATTAACGGAATTGATTCACGGCAAGCACGTGATAAGGCGAGACATGCTCGTACTC

AGTGGCATGGAGTAGATACTCATGATCCAAATACAGCTAGGAAGTGCGTGATGCAAGCTGTTGAGCAGTC

TGCGAAAGGTGAAATATATGCTGCTCTTGCTTTATTAGACCTTGTATCTAACAACGATGCAAACATGCAG

GTCAATGAGGCGGGGCATACTATTTTGCATCTAGCAGGTATTGAAGGTAGTGACCCAGCTTTCACTGAAG

TCCTTCTTCTGAAGGGTTGTGCTTTAAATACTAGGGATATAAATGGTAATACTCCATTACATACAATTGC

GGCTACGGTAGGAAAAAACACTTTAGGCAATCTTGATGCTATATGTGACGGAGCTCTTATAGCAGATGTT

AATGCTAAGAACAACGAGGGAGACACTCCACTGCATATTGCTACGAAGCGTATGGATCACGAGAAAATAG

ACGCTCTTCTCTCGAGGCTTAGCGATATTAGCGTGGCAAATAATGCTGGGAAAACCGTTTTCCACATCGT

TGCAGAGCGCTGGCCAAGGCGAAATGTTTTAGCATACATTGATAAAGTGCAAGGAGCGGTATCGCCAAAT

ATTGAGGGCAATCGCGAGTGTGCAGAGGCACTAATATTCCCGGATCAAGAAGGGATRAGTGCAGTGCAGC

ATATTATTAGAAGGAATGTACCAGATGCTGGGAAGATCTTCGAGASAGCTCTTAGAATTGCGGATAAAGT

GTACAGCTCAGGTTCCCCAGAAGTAAGATCTCTCTTCACRTGCCCTGGTATTAAAGATGCAAAAACGCTA

CTGCATTTAGYGTCTTCTAATGATAGCAAAGATTTTAATCGTACTGCGAKAATAATAGTAGAAGAAGCTY

RTCATAGGTTTGGAGAAGAACCTTTTACTCATGTCGACATTTTCGGTAATGCACCTATACA

Start codon

Sequencing primers

Substitutions
